# Machine learning unveils surface refractive index dynamics in comb-like plasmonic optical fiber biosensors

**DOI:** 10.1038/s44172-024-00181-9

**Published:** 2024-02-23

**Authors:** Hadrien Fasseaux, Médéric Loyez, Christophe Caucheteur

**Affiliations:** 1 Electromagnetism and Telecommunications Department, Umons, 31, Boulevard Dolez, Mons, 7000 Belgium; 2 Proteomics and Microbiology Department, Umons, 6, Avenue du Champ de Mars, Mons, 7000 Belgium

**Keywords:** Scientific data, Biological techniques, Optical sensors, Imaging and sensing

## Abstract

The precise measurement of surface refractive index changes is crucial in biosensing, providing insights into bioreceptors–analytes interactions. However, correlating intricate spectral features, with these refractive index variations remains a persistent challenge, particularly in optical fiber gratings-based Surface Plasmon Resonance sensing. Here, we introduce a machine learning-based approach to address this ongoing issue. We integrate a regression model with gold-coated tilted fiber Bragg grating sensors. This enhances signal stability and precision, enabling a correlation between spectral shifts and refractive index changes. Our approach eliminates the need for individual sensor calibration, thereby bolstering the effectiveness and efficiency of the sensing layer. We demonstrate the model’s versatility by showcasing its efficacy across two data acquisition systems with different resolutions, allowing for comparative analysis and robustness enhancement. Its application in a biosensing experiment for insulin functionalization and detection, demonstrates how this breakthrough approach marks an advancement in real-time refractive index monitoring.

## Abstract (web summary)

Fasseaux and co-authors present an enhanced performance of the tilted fiber Bragg grating-based biosensor. Using regression-based machine learning, the changes in the refractive index via changes in the spectral shift can be monitored in real-time.

## Introduction

Surface refractive index (RI) variation plays a crucial role in the realm of biosensing, offering valuable insights into the complex interplay between bioreceptors and their corresponding analytes^1–4^. Surface plasmon resonance (SPR) has emerged as a highly-responsive technique for monitoring biomolecular interactions, owing to the RI variations occurring in proximity to a metal surface upon biomolecule adsorption^5–9^.

In recent years, a diverse range of physical platforms grounded in SPR principles, such as the Kretschmann prism or optical fibers, have been extensively explored, leading to the development of commercially viable devices^10–16^. Optical fiber-based platforms offer a compelling array of attributes, including immunity to electromagnetic interference, lightweight and compact design, cost-efficiency, wavelength multiplexing capacity, and adaptability^17–20^.

**Fig. 1:**
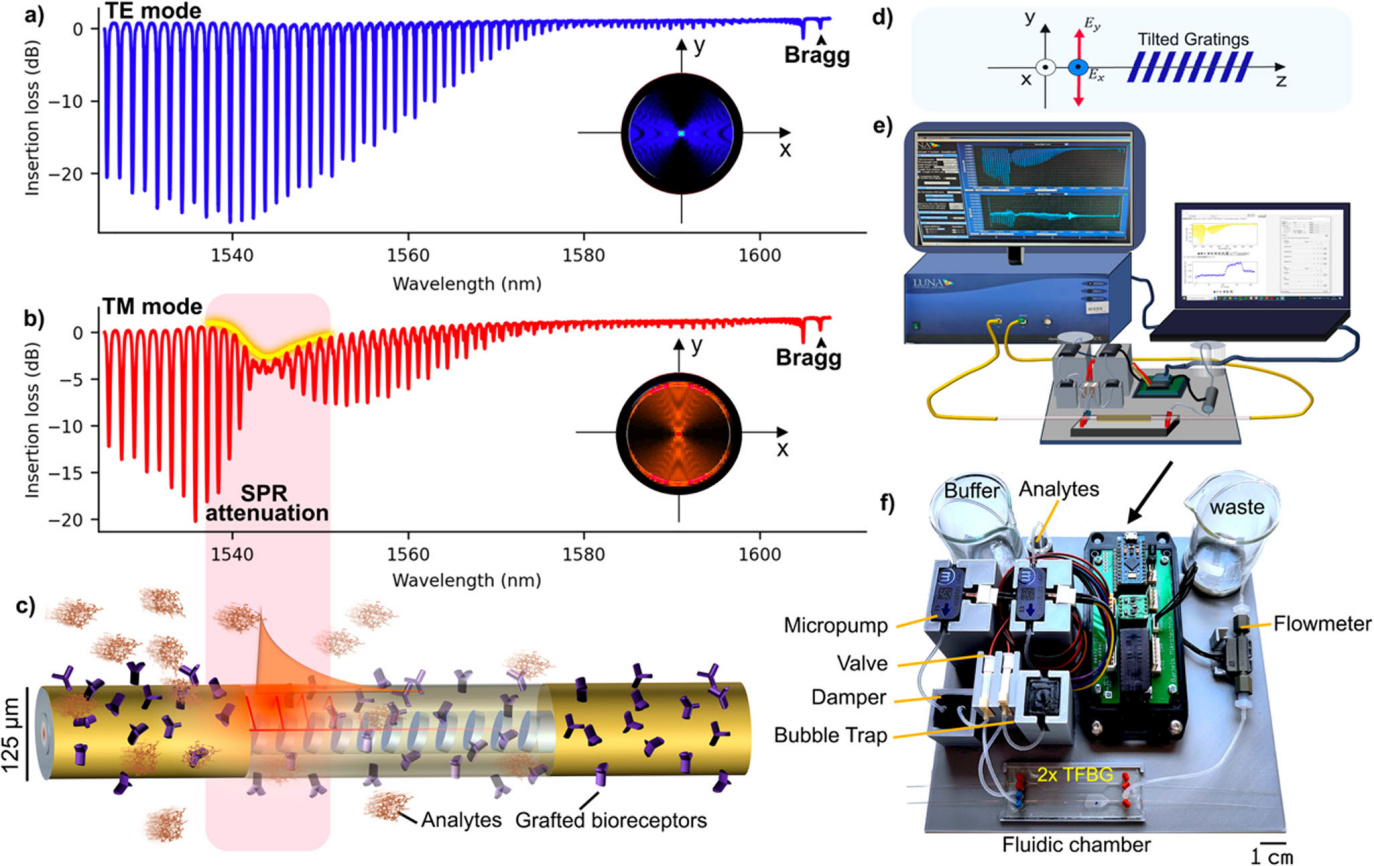
The general concept associated with the utilization of gold-coated tilted fiber Bragg grating (TFBG) biosensors is depicted. **a** The insertion loss spectrum is monitored with incident light in the transverse electric (TE) mode, and (**b**) in the transverse magnetic (TM) mode. **c** The biosensor features a telecommunication-grade fiber containing an inclined grating at its core. The cladding surface is coated with gold, and receptors (depicted in purple) specific to the analytes (depicted in orange) are affixed thereto. **d** The surface plasmon resonance mechanism is facilitated by the presence of TFBGs located inside the core along the fiber axis (z-axis), coupled with its excitation through the TM mode (electric field oscillates along the y-axis). **e** The experimental setup includes a fiber sensor connected in transmission with an optical vector analyzer. **f** A microfluidic system, comprising micropumps, valves, a damper, and a bubble trap, is employed to maintain a steady flow at 30 μg mL^−1^ into the microfluidic chamber, where the sensor is sealed.

Among this array of configurations, gold-coated tilted fiber Bragg gratings (Au-TFBG) emerge prominently, distinguished by their temperature compensation ability^21^. Furthermore, their straightforward fabrication not only preserves the inherent optical properties of the single-mode fiber platform but also enables the extraction of cladding modes involved in core mode coupling, achieved through a single spectral measurement^22–26^. These cladding modes are illustrated in Fig. 1a through the insertion loss spectrum (ILs) associated with transverse electric (TE) polarization. When operating in the transverse magnetic (TM)-polarization mode^27^, this dense spectral comb reveals a distinctive notch centered around the resonance wavelength, signifying the region of highest sensitivity (see Fig. 1b).

In response to these spectral changes prompted by a shift in RI, several demodulation techniques have been conceived, linking shifts in wavelength or amplitude to corresponding RI alterations^23,28–32^. Understanding the evolution of the spectrum, characterized by distinct peaks in the spectral comb, is paramount. Each mode undergoes a sigmoidal evolution, with the region of highest sensitivity confined to a specific range of refractive indices^28^. Challenges arise when observing RI changes over extended intervals and transitioning smoothly between modes during analysis. However, certain methodologies, such as the cross-demodulation method (refer to “Spectral shape characteristics”), mitigate the impact of such mode transitions.

Furthermore, recent investigations by Chubchev et al.^33^ have laid the groundwork for integrating machine learning methodologies in the analysis of spectral data derived from a plasmonic fiber sensor utilizing a TFBG.

Their work notably emphasizes the implementation of an algorithm trained on one fiber and tested on another, particularly in the context of a minor RI shift. It is crucial to underscore that their efforts have demonstrated the viability of a machine learning algorithm for the Au-TFBG sensor (schemed in Fig. 1c), achieving a level of precision that would have been challenging to attain through alternative means.

However, establishing a correlation between alterations in the spectral pattern and RI fluctuations remains challenging due to the nature of the spectral changes^28^. The quantification of sensor sensitivity through index solutions becomes therefore imperative, despite potential implications for the sensor’s efficacy in practical biodetection contexts. This underscores the necessity of developing a proficient model endowed with the capability to correlate measured spectral fluctuations with corresponding RI modulations, leading to time savings, heightened reliability, and streamlined medium characterization.

In this study, we present a pioneering approach to address the challenges posed by intricate spectral patterns, enabling real-time tracking of RI variations in biosensing assays. Through the integration of a regression-based machine learning model, we establish a robust correlation between spectral fluctuations and changes in RI within an external medium. Furthermore, we validate the efficiency of this method across two interrogation devices, one of which is the most extensively employed instrument in this research field. This innovative approach streamlines biosensing protocols by obviating the need for a dedicated sensor sensitivity calibration step and ensures the preservation of the effectiveness and efficiency of the sensing layer. Importantly, it leads to a reduction in experimental duration, marking a substantial advancement in the field. Additionally, for the first time, we introduce real-time monitoring of RI changes during biosensing trials. This breakthrough overcomes the longstanding challenge that has hindered sensor comparability.

## Results

### Spectral shape characteristics

**Fig. 2:**
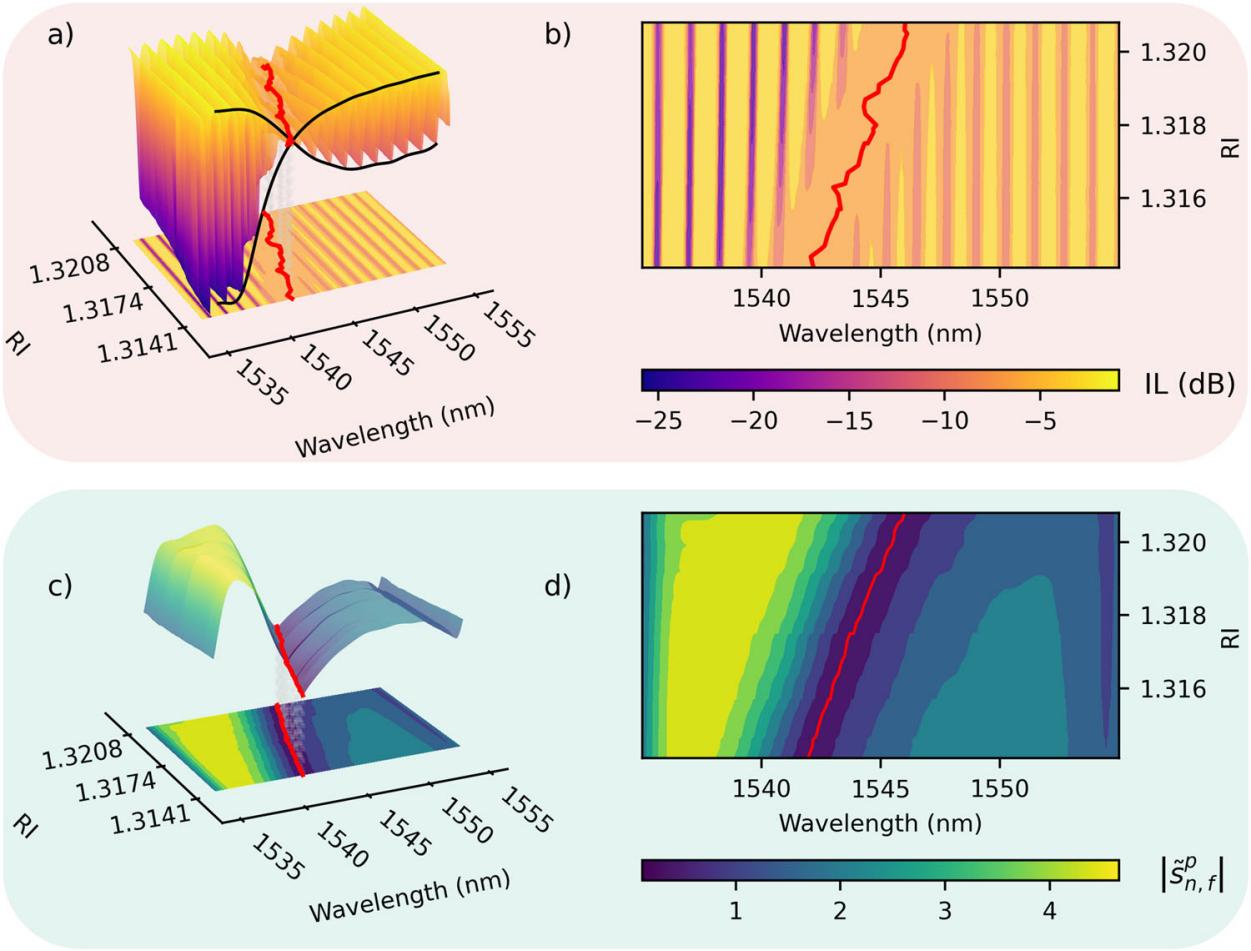
Experimental transmission spectra for various surrounding refractive index (RI) media of a gold-coated tilted fiber Bragg grating (TFBG). **a** Spectral evolution of the insertion loss (IL) with increasing RI. The attenuation region within the IL spectrum covers a span of 3 nm. The point of intersection of the cubic interpolations, represented by black lines, offers an approximate visualization of the SPR phenomenon emphasized through its projection on the refractive index-wavelength plane (**b**). The progression with RI is also illustrated through filtered transmission spectra (**c**). Notably, the linear trajectory of the local minima within the SPR attenuation zone is evidenced by the corresponding projections on the refractive index-wavelength plane (**d**). This near-linear evolution displays a sensitivity akin to theoretical predictions (~605.53 nm RIU^−1^ in this specific case).

As aforementioned, the incident polarization state plays a crucial role in the spectral shape of the Au-TFBG^34^. Two distinct polarization states, known as TM and TE (also known as p and s), give rise to different spectra (see Fig. 1a, b). The TM-polarized state exhibits a comb-shaped spectrum with attenuation associated with SPR, while the TE-polarization, orthogonal to the TM-polarization, does not experience such attenuation. The resonance wavelength of a surface plasmon is determined by the RI of the surrounding dielectric material^22^ as

1 $${\lambda }_{{{{{{{{\rm{sp}}}}}}}}}={{{{{\rm{Re}}}}}} \left({n}_{{{{{{{{\rm{eff}}}}}}}}}^{{{{{{{{\rm{co}}}}}}}}}+\sqrt{\frac{{\epsilon }_{{{{{{{{\rm{m}}}}}}}}}{\epsilon }_{{{{{{{{\rm{d}}}}}}}}}}{{\epsilon }_{{{{{{{{\rm{m}}}}}}}}}+{\epsilon }_{{{{{{{{\rm{d}}}}}}}}}}}\right)\frac{{\Lambda }_{{{{{{{{\rm{g}}}}}}}}}}{\cos \theta },$$

where, Λ_g_ represents the grating period, *θ* is the tilt angle of the grating with respect to the cross-section plane (see Fig. 1d). *ϵ*_m_ and *ϵ*_d_ are the dielectric constants of the metal and dielectric medium, respectively, and ${n}_{{{{{{{{\rm{eff}}}}}}}}}^{{{{{{{{\rm{co}}}}}}}}}$ denotes the effective RI of the core mode. Within a RI range of 0.01 refractive index units (RIU) starting from the water RI, the resonance wavelength exhibits a linear relationship with a variation of ~567 nm RIU^−1^. However, determining the resonance wavelength precisely is challenging due to the intricate nature of the attenuation area within the ILs, as shown in Fig. 2a depicting the SPR behavior under different surrounding refractive index (SRI) media.

The attenuation zone spans 3 nm with minimal amplitude variation relative to other wavelength ranges, hindering the precise determination of the SPR wavelength. To overcome this challenge, a cross demodulation method can be employed, which involves tracking the intersection of two interpolations, as depicted by the black crossed curves in Fig. 2a. This approach allows for the approximation of the SPR with an evolutionary trend exhibiting a sensitivity comparable to that of the SPR (~575.54 nm RIU^−1^ in this case). Nevertheless, a limitation of this approach arises from the impact of mode transition on the linear response of the observed intersection point which introduces complexity in accurately determining the SPR wavelength, as depicted in Fig. 2b. To address this limitation, integration of a band-pass filter within the generalized frequency domain is proposed. The results depicted in Fig. 2c demonstrate that implementing such filtering for ILs leads to a distinct local minimum, easy to track, and exhibiting a more linear progression with increasing RI (red line). Nevertheless, the observed degree of linearity is contingent upon the portion of the generalized frequency that remains after filtering. By training a machine learning model on spectra obtained from multiple fibers, we can ascertain the optimal filtering approach and also establish the linear correlation between the evolution of this minimum and SRI variations.

### Machine learning model

We have devised a machine learning model with the aim of predicting the RI change of the fluid surrounding an Au-TFBG. The model utilizes the insertion loss spectra corresponding to multiple input polarization states, leading to resonance with a surface plasmon. This approach enables the establishment of a predictive relationship between the observed spectral features and the SRI variations.

#### Data acquisition and preprocessing

The optical vector analyzer (OVA), specifically the OVA CTe 4000NF from Luna Technologies Inc. (set up as schematically illustrated in Fig. 1e and described in “Experimental setup with the OVA”), played a crucial role in our data acquisition process. It provided essential elements of the Jones matrix (${\mathbb{J}}(\lambda )\in {{\mathbb{C}}}^{2\times 2}$) characterizing the system under test, enabling precise determination of insertion loss spectra for various input polarization states. Indeed, let us consider an elliptical state $\left\vert a\right\rangle$ defined with the Dirac notation as

2 $$\left\vert a\right\rangle =\left(\begin{array}{c}\cos \alpha \cos \epsilon -i\sin \alpha \sin \epsilon \\ \sin \alpha \cos \epsilon +i\cos \alpha \sin \epsilon \end{array}\right),$$

where *α* ∈ [0, *π*] is the orientation of the major axis of the ellipse with respect to the x-axis, and *ϵ* ∈ [ − *π*/4, *π*/4] denotes the ellipticity. As such a state $\left\vert a\right\rangle$ is transmitted through the system, it undergoes alterations, resulting in the emergence of a new state $\left\vert b(\lambda )\right\rangle ={\mathbb{J(\lambda )}}\left\vert a\right\rangle$ at the output. The corresponding ILs can be obtained by taking the logarithm of the relationship

3 $$I(\lambda )=\frac{\langle b(\lambda )| b(\lambda )\rangle }{\langle a| a\rangle }=\frac{\langle a| {{\mathbb{J}}}^{{{{\dagger}}} }(\lambda ){\mathbb{J}}(\lambda )| a\rangle }{\langle a| a\rangle },$$

where ${{\mathbb{J}}}^{{{{\dagger}}} }(\lambda )$ denotes the Hermitian conjugate of ${\mathbb{J}}(\lambda )$.

To determine the optimal input polarization state resulting in the TM-mode spectrum, the initial step involves identifying the pair of input states that produces its orthogonal TE-mode, a comparatively straightforward task. Specifically, the focus lies on determining the polarization states that minimize the average of the output ILs, yielding the two angles (*α*, *ϵ*)_TE_ corresponding to the TE-mode spectrum. Subsequently, the TM-mode is obtained by applying the transformations *α* → *α* ± *π*/2 and *ϵ* → − *ϵ*. So, the measurement of a fiber sensor in a known RI solution for an input polarization state, respectively represented by the indices *f*, *n,* and *p* = 1, constitutes the dataset ${{{{{{{\bf{S}}}}}}}}=\{{s}_{n,f}^{1}\}$. However, even when a state close to the TM-input state is employed, attenuation is still present.

**Fig. 3:**
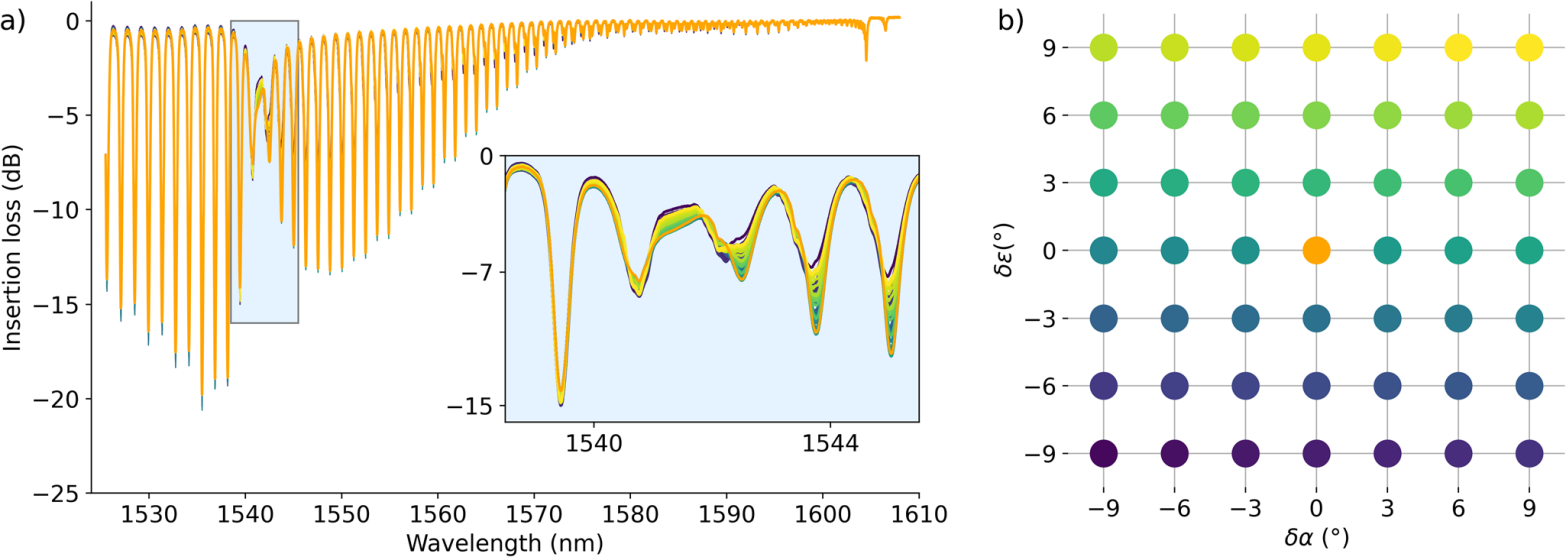
Polarization dependency of the insertion loss spectrum. **a** Insertion loss spectra of a distinctive optical fiber immersed in a refractive index solution with a precisely measured value of (1.3337 ± 0.0002) RIU at 589 nm employing 49 diverse input polarization states. The inset emphasized a detailed zoom on the surface plasmon resonance notch. **b** Displays a colormap representing each of the 49 utilized input polarization states. The highlighted orange spectrum is obtained through the methodology detailed in “Data acquisition and preprocessing”, whereas others represent variations achieved by changing the orientation (*δα*) and ellipticity (*δϵ*).

Data augmentation becomes imperative to enhance the robustness of our model. By recording ILs for input states in close proximity to the TM input state, we ensure a more comprehensive dataset for training, even in the presence of attenuation (see corresponding Ils in Fig. 3a for the 49 polarization state in Fig. 3b). For instance, considering the pair (*α*, *ϵ*)_TM_ that yields the ILs of the TM-mode, we also obtain the notch for input states (*α* ± *δα*, *ϵ*±*δϵ*)_TM_, where *δα* and *δϵ* are small (chosen from the set 0°, 3°, 6° in our case). After this data augmentation, the data set serving as the basis for training the model is ${{{{{{{\bf{S}}}}}}}}=\{{s}_{n,f}^{p}\}$, where *p* denotes a polarization state leading to ILs notch.

#### Model architecture

Our model is grounded in spectral transformations. Each spectrum, derived from fibers immersed in various RI solutions and subjected to diverse input polarizations, undergoes a Fourier transformation, followed by band-pass filtering and an inverse Fourier transformation. Local minima are identified, and the wavelength difference between these minima and those of a reference spectrum is measured. RI changes serve as labels for training. A linear function correlating these measured wavelength changes and the associated RI variations is established, providing a quantitative relationship. The evaluation metric employed is the mean absolute error (MAE).

#### Model training

The acquired spectra are Fourier transformed to highlight their generalized frequency decomposition, given by

4 $${{{{{{{\mathcal{F}}}}}}}}[{s}_{n,f}^{p}(\lambda )]={{{{{{{\mathcal{F}}}}}}}}{s}_{n,f}^{p}(\nu )={\int}_{{\mathbb{R}}}{e}^{i2\pi \lambda \nu }{s}_{n,f}^{p}(\lambda )d\lambda ,$$

where *ν* are the generalized frequencies. Then, a filter function in the generalized frequency space is implemented using a super-Gaussian of degree 4, defined as:

5 $$\begin{array}{r}f(\nu ;\mu ,\sigma )={e}^{-{\left(\frac{\nu -\mu }{2\sigma }\right)}^{4}}.\end{array}$$

The transformed spectrum ${{{{{{{\mathcal{F}}}}}}}}{s}_{n,f}^{p}(\nu )$ is multiplied by the filter function, and the inverse Fourier transform is finally performed on the result, yielding ${\widetilde{s}}_{n,f}^{\,p}$. Through careful selection of the variables *μ* and *σ*, the resulting spectrum modulus exhibits a local minimum ${\lambda }_{\min }$ around the predicted SPR wavelength, as depicted in Fig. 2c. This local minimum exhibits a linear trend with increasing RI, as highlighted in Fig. 2d.

**Fig. 4:**
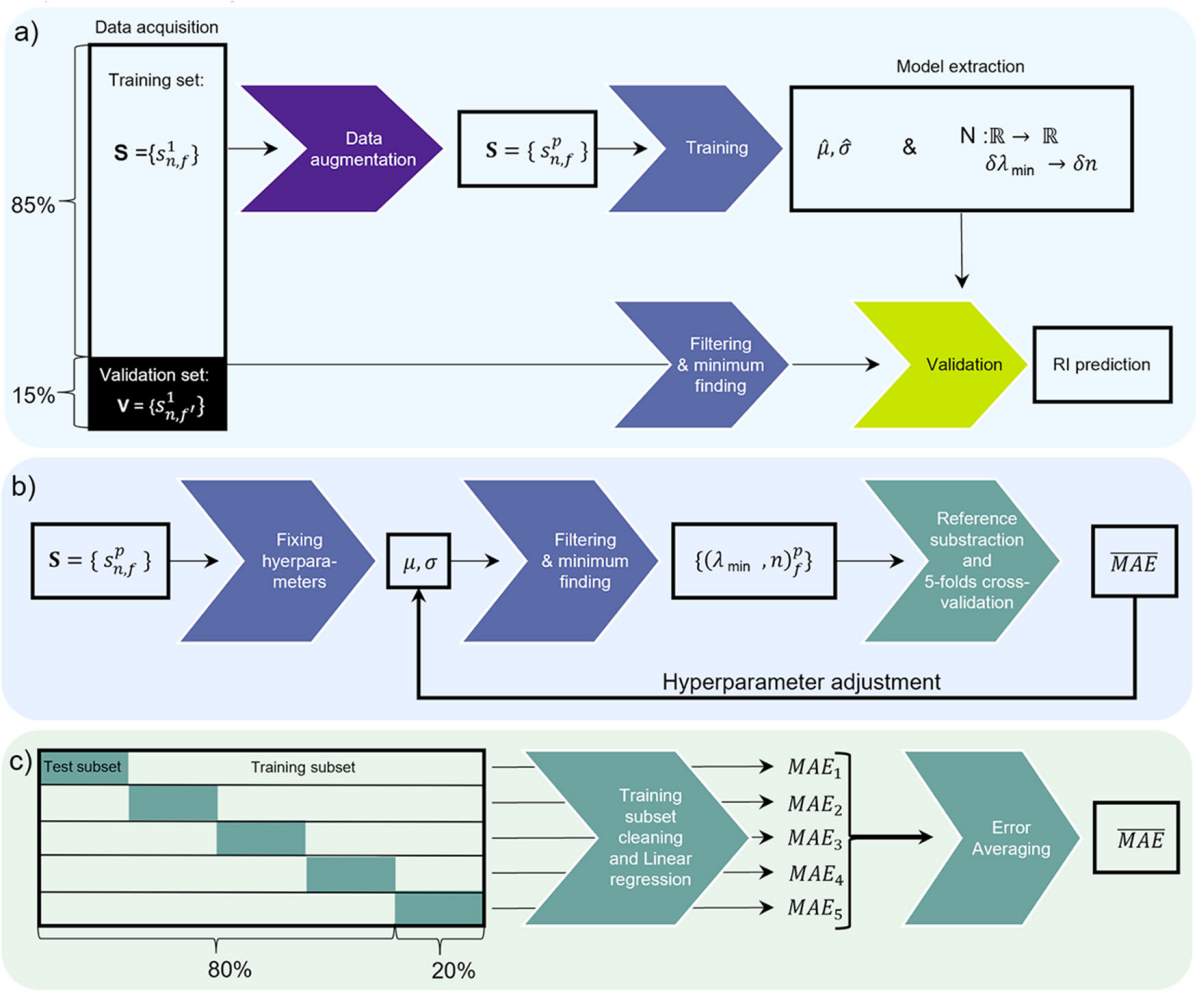
Flowchart outlining the proposed method. Rectangles represent input and output data, while chevron arrows indicate the execution steps. The model focuses on optimizing the hyperparameters (*μ* and *σ*) of the band-pass filter to achieve a linear relationship, N, between minimum wavelength changes (*δλ*) and refractive index (RI) changes (*δn*) in the filtered spectrum. **a** Expansion of the training set, comprising 470 spectra from 12 different fibers (i.e.*f* ∈ [1, 12]), by incorporating input polarization states resulting in SPR attenuation within the insertion loss spectrum (ILs). Training on this expanded spectrum enables the determination of optimal hyperparameters ($\hat{\mu }$ and $\hat{\sigma }$) and the development of a linear model that correlates the evolution of the local minimum in the filtered ILs with the RI evolution. **b** The training process entails identifying hyperparameters that minimize the average mean average error (MAE) through linear regression. **c** The cross-validation process for model evaluation and performance assessment.

The machine learning model (see Fig. 4a) optimizes two hyperparameters, *μ* and *σ*, of a band-pass filter to extract a linear correlation between SRI changes (*δn*) and the corresponding minimum wavelength change (*δλ*). To obtain the linear model $N(\delta \lambda ;\hat{\nu },\hat{\sigma })$ that correlates the wavelength evolution *δλ* to SRI shift *δn* with optimized hyperparameters $\hat{\mu }$ and $\hat{\sigma }$, we employ the least squares method in conjunction with a fivefold cross-validation procedure.

The training dataset consists of pairs ${({\lambda }_{\min },n)}_{f}^{p}$, where ${\lambda }_{\min }$ is the local minimum, *f* and *p* denote the utilized sensor and the input polarization state, respectively. For each fiber *f*, we determine the pair ${({\lambda }_{\min },n)}_{f}^{p}$ and calculate the variations in both the RI and the associated minimal wavelength by subtracting the value of the pair obtained while the fiber is immersed into water (see Fig. 4b). Thus, we derive the pairs ${(\delta {\lambda }_{\min },\delta n)}_{f}^{p}$. These datasets are shuffled and divided into five equally sized segments, with one segment serving as the test subset and the remaining four segments as the training subset (see Fig. 4c). To ensure independence between training and testing, we exclude ${(\delta {\lambda }_{\min },\delta n)}_{f}^{p}$ values from the training subset if the corresponding *n* and *f* indices also appear within the training subset. This prevents training and testing on the same fiber exposed to the same RI conditions. Then, we apply the least squares method to determine the function $N(\delta {\lambda }_{\min };\nu ,\sigma )$. Subsequently, this function is applied to the test subset data, and the MAE is calculated. The test subset is then exchanged with one of the four training subsets, and the process is repeated five times, covering all constituent parts of the training set as the test subset. This results in five measured MAEs, which are then averaged. The training procedure is repeated with various *μ* and *σ* hyperparameter values to minimize the average MAE. A linear regression is finally performed on the entire test set to obtain the model $N(\delta {\lambda }_{\min };\hat{\mu },\hat{\sigma })$. The flowchart in Fig. 4 summarizes the machine learning approach that we have implemented.

#### Model validation

**Fig. 5:**
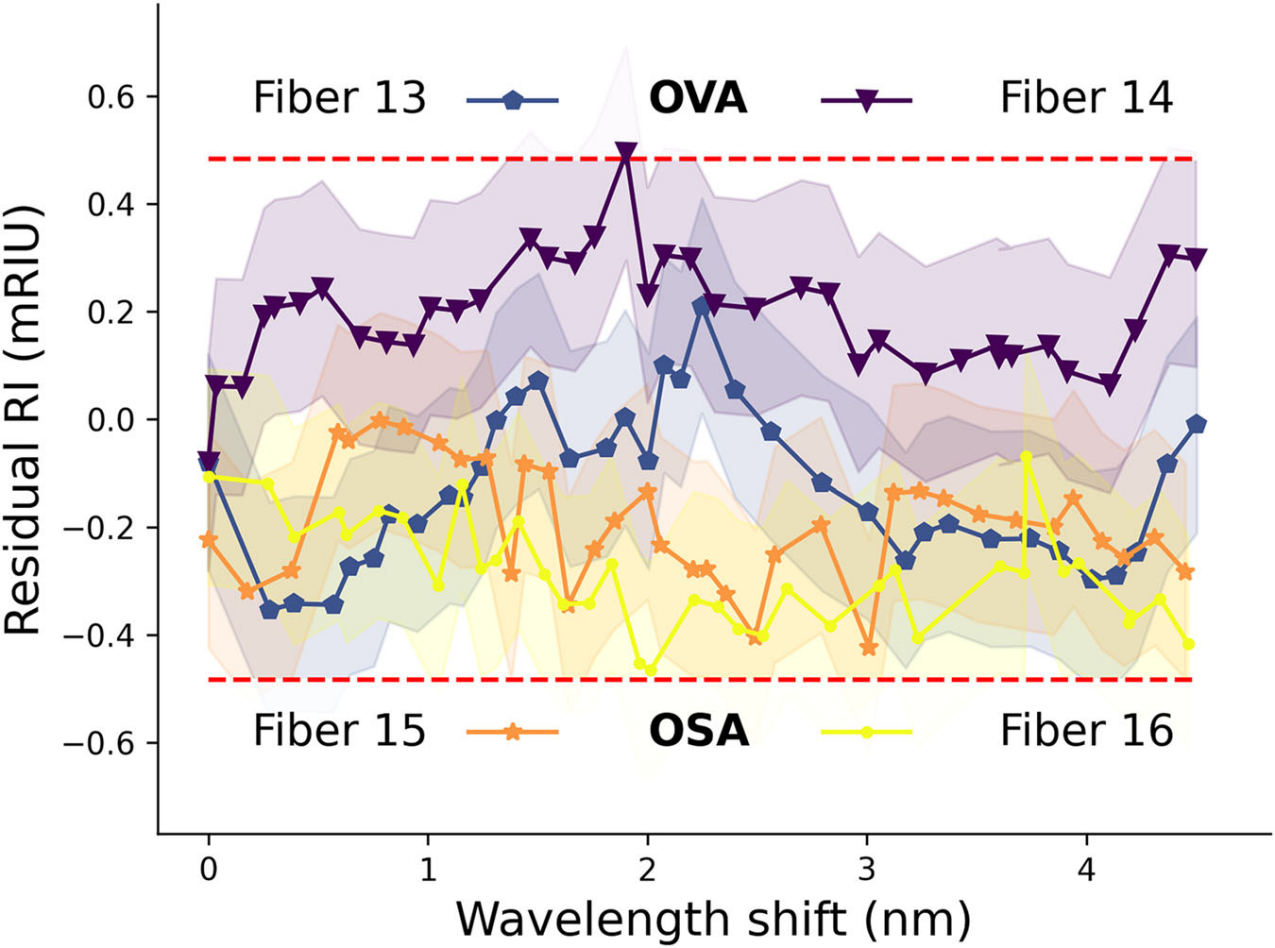
Validation curves illustrate the disparity between experimentally determined refractive index values and model predictions. Four separate fibers were used for refractometry measurements using and optical spectrum analyzer (OSA) and an optical vector analyzer (OVA). Each measurement is shown within the refractometer tolerance zone (±2 nm). Red dashed lines delimit the 95% confidence interval of the model.

The model validation is conducted on an independent and unused dataset originating from four distinct fibers, designated as fibers 13 through 16, as illustrated in Fig. 5. Two of them were interrogated with the OVA (fiber 13 and 14) and the two others were monitored with an optical spectrum analyser (OSA) based setup (see “Experimental setup with the OSA”) to show the applicability of the method to equipment with lower spectral resolution (50 pm for the OSA and 2.4 pm for the OVA). After subjecting the ILs of a single TM-polarization to the aforementioned filtering process, we identify the local minimum variation $\delta {\lambda }_{\min }$. These shifts serve as inputs for the model $N(\delta {\lambda }_{\min };\hat{\mu },\hat{\sigma })$, which predicts the corresponding RI denoted by $\hat{n}$. The results of the residual RI (i.e. the difference between the measured value and the predicted one), along with a 95% confidence interval, are depicted in Fig. 5.

In summary, our model relies on a spectral processing architecture, extracting relevant features to predict RI changes in immersed optical fibers. The parameters of the obtained band-pass filter are $\hat{\mu }=0.799\,{{{{{{{{\rm{nm}}}}}}}}}^{-1}$ and $\hat{\sigma }=0.097\,{{{{{{{{\rm{nm}}}}}}}}}^{-1}$. The relationship between RI change *δn* and wavelength change *δλ* is measured at 0.00169474 RIU nm^−1^.

### Biosensing application

**Fig. 6:**
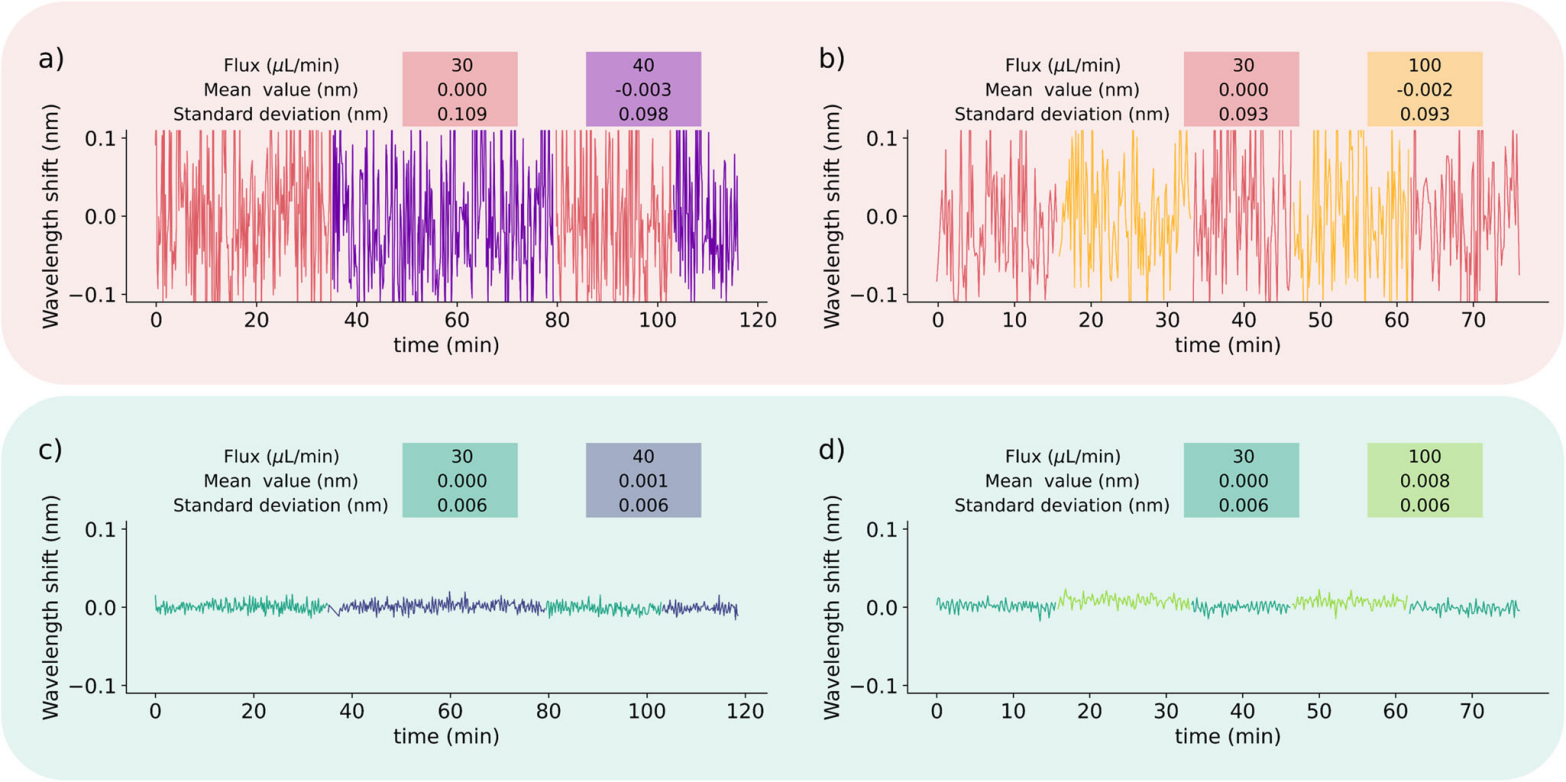
Signal stability of a gold-coated tilted fiber Bragg grating immersed in a buffer solution across flow rates of 30, 40, and 100 μL min^−1^. Investigation involves examining both fluctuations (**a**, **c**) and flow transitions (**b**, **d**) using both the cross-demodulation method (**a**, **b**) and the proposed technique (**c**, **d**). The correlation function reveals a standard deviation of 10^−5 ^RIU, with mean values of 10^−6 ^RIU and 13 × 10^−6 ^RIU for flow rates of 40 and 100 μL min^−1^, respectively.

Biosensing experiments were conducted with the OSA and a dedicated microfluidic system (refer to “Experimental setup for biosensing” and Fig. 1f). The latter is employed to uphold a consistent flow and prevent sudden changes that could impact the baseline. As illustrated in Fig. 6a, c, minor alterations in flow do not influence either the baseline or dispersion. However, abrupt changes in flow, especially at higher levels as depicted in Fig. 6b, d, do affect the signal’s baseline. This perceptibility of this alteration is exclusively discernible through the proposed method, resulting in a less dispersed signal (standard deviation around 0.006 nm) compared to the cross-demodulation method (standard deviation around 0.1 nm). Utilizing the proposed method and the correlation function reveals a baseline shift of 1.3 × 10^−5 ^RIU during a transition from a flow of 30–100 μL min^−1^. However, the dispersion remains constant at 10^−5 ^RIU. To mitigate this undesired shift, a microfluidic system is employed to maintain a steady flow. In this regime, any change approximately three times greater than the maximum dispersion can be deemed measurable, establishing a detection limit of 3 × 10^−5 ^RIU. The target (or the analyte) is insulin diluted at a concentration ranging from 0.1 to 1 μg mL^−1^.

#### Biofunctionalization

**Fig. 7:**
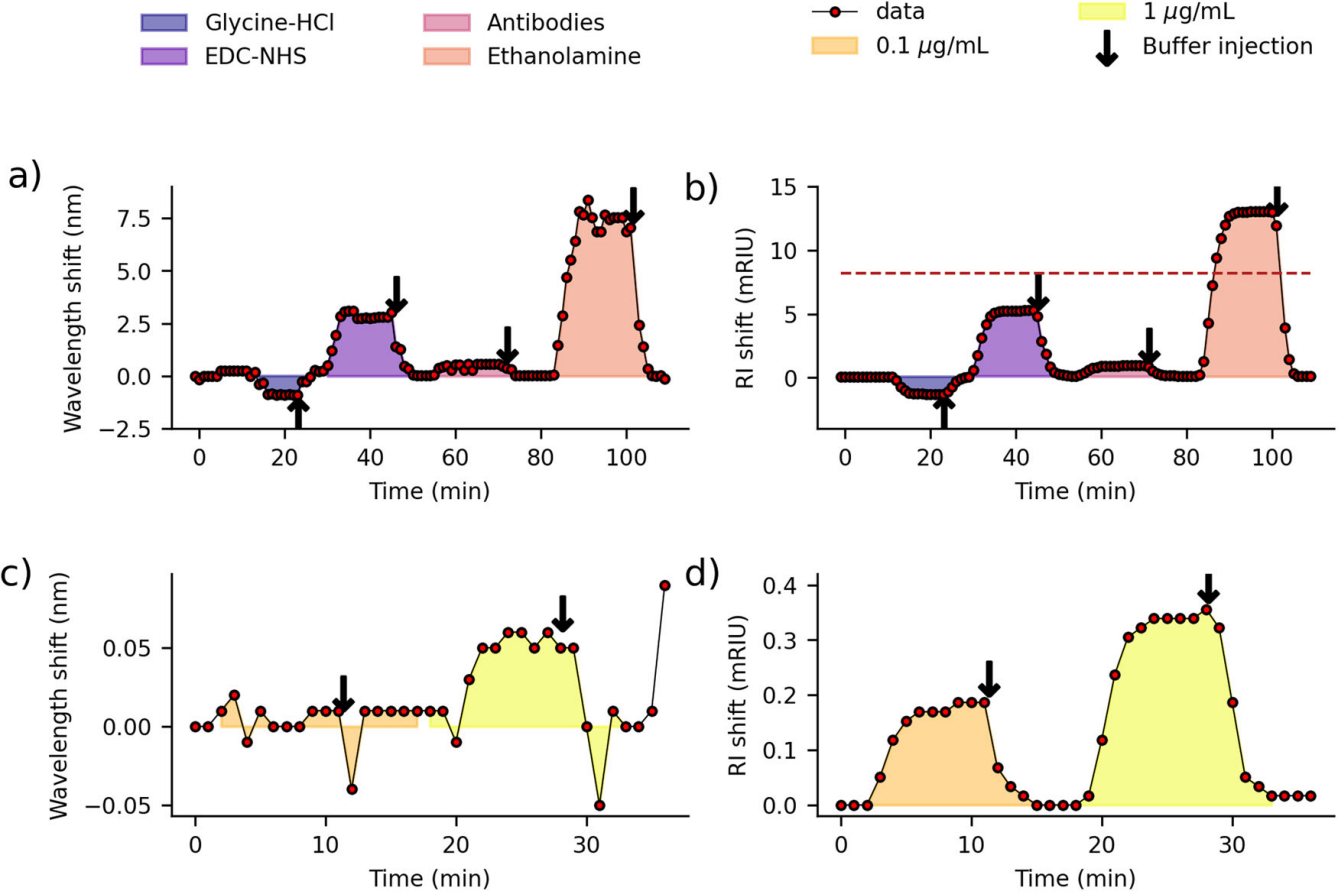
Biosensing results. **a**, **b** Functionalization process: a continuous flow of 30 μL min^−1^ of various solutions is injected into the microfluidic chamber containing the sealed gold-coated tilted fiber Bragg grating (TFBG). This process leads to the attachment of anti-insulin antibodies as receptors. The results are analyzed using the cross-demodulation method (**a**) and the proposed model (**b**). The red dashed horizontal line denotes the refractive index boundary for which the model was trained (1.3432, corresponding to an 8.2 mRIU shift relative to the buffer reference). Biodetection process (**c**, **d**): 2 insulin solutions at concentrations of 0.1 μg mL^−1^ and 1 μg mL^−1^, are introduced successively into the system. **c** displays it using the cross-demodulation technique, while (**d**) is the result of our proposed method.

**Fig. 8:**
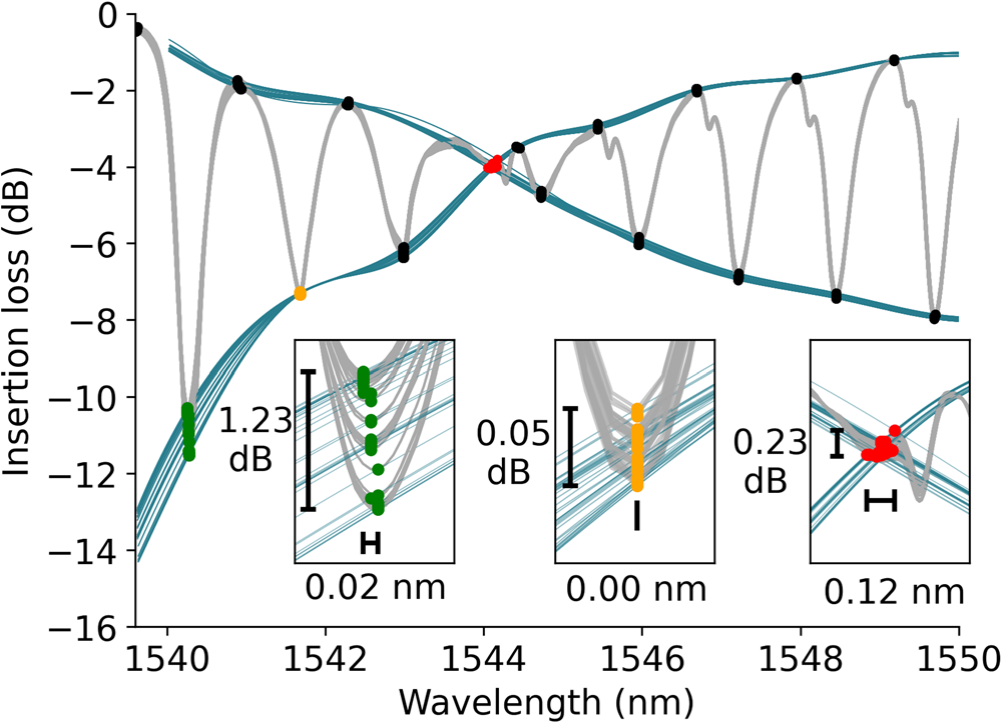
Cross demodulation method. The evolution of insertion loss spectra during biodetection process emphasizes the cross-demodulation technique and the variation difference between peaks.

Developing a biosensor from an Au-TFBG necessitates the grafting of receptors on its sensing surface. The initial steps involve binding self-assembled monolayer (SAM) by immersion in a solution containing 11-mercaptoundecanoic acid (95% purity, Sigma-Aldrich) diluted in absolute ethanol for a duration of 18 h. Subsequently, the surface undergoes cleaning with a pH 1.5 Glycine-HCl solution. Activation of the carboxyl terminated SAM, generating reactive succinimide esters facilitated by a mixture of N-hydroxysuccinimide (NHS) and 1-ethyl-3(3-dimethylaminopropyl) carbodiimide (EDC). Mouse anti-human insulin antibodies are then introduced over the sensing surface, forming a covalent linkage between amine group of the antibodies and the matrix through the esters. To complete the functionalization process, any remaining active esters are deactivated using ethanolamine-HCl. Throughout the procedure, buffer rinsings, employing a mixture of HEPES (4-(2-hydroxyethyl)-1-piperazineethanesulfonic acid), NaCl, (ethylenediaminetetraacetic acid), and P20 surfactant, are performed between each solution application. Figure 7 illustrates all the stages excluding the 18-h SAM deposition procedure, comparing the cross-demodulation technique (Fig. 7a) with the proposed method (Fig. 7b), emphasizing the much enhanced fidelidy achieved with our approach. Table 1 consolidates RI changes relative to the buffer’s RI (1.3351 at 589 nm) and the values predicted by the model. While the sensor immersed in ethanolamine exhibits a spectrum that was neither utilized for training nor validation, the predictions align with the measured values, thereby showing the potential of our model for RI changes beyond those encountered during training. The red dashed horizontal line in Fig. 7b denotes the threshold beyond which collected spectra were not employed for training or validation purposes. The progression of ILs throughout the detection process is illustrated in Fig. 8, with the intersection point highlighted in red, utilizing the cross demodulation technique (represented by blue curves). The supplementary views focus on two peaks (colored green and orange) situated towards the shorter wavelength side of the SPR notch, underscoring the distinction in variation (amplitude and wavelength) between the peaks.

**Table 1:** Comparative table presenting the values of measured and predicted refractive index (RI) shifts

Solutions	Measured RI shift (mRIU)	Predicted RI shift (mRIU)
Glycine-HCl	−1.4 ± 0.4	−1.29 ± 0.48
EDC-NHS	+5.1 ± 0.4	+5.28 ± 0.48
Ethanolamine	+13.6 ± 0.4	+13.04 ± 0.48

The reference solution is the buffer with a RI of 1.3351 (measured at 589 nm).

#### Biodetection

In a sequential way, we introduced two insulin solutions with distinct concentrations (0.1 μg mL^−1^ and 1 μg mL^−1^) into the system, with careful buffer rinsing between each injection. The experimental results of this entire process are depicted in Fig. 7 using the cross-demodulation method (Fig. 7c) and the proposed one (Fig. 7d). Clearly, the curve demonstrates the demodulation’s capability to operate within the sub-mRIU range of shift. The observable signal shift before and after analyte injection highlights the sensor’s suitability for biodetections. Notably, the binding dynamics assume a pivotal role, often comparable in significance to changes in buffer levels. While this article’s primary focus lies beyond a detailed exploration of these dynamics, the curves prominently indicate the method’s potential for future analytical investigations.

## Discussion

The presented approach provides a practical solution for addressing the challenges associated with extracting valuable information from the ILs of an Au-TFBG-based biosensing platform during biosensing experiments. The proposed regressive machine learning model was trained on an extensive dataset comprising 23,030 spectra from 12 diverse Au-TFBG sensors, encompassing 470 refractometric experiments and 49 different polarization states. By relying on filtering the ILs within the generalized frequency domain, the training process involves the determination of optimal filter parameters, leading to a simplified spectrum compared to the dense spectral comb one. Additionally, it establishes the relationship correlating spectral changes with RI variations. The training spectra were acquired using an OVA, and we demonstrate the method’s applicability to an optical spectrum analyzer, a widely utilized instrument in this research domain. Results from four additional, previously unseen Au-TFBG sensors validate the robustness of the correlation, highlighting the method’s effectiveness and versatility. Predictions fall within a 95% confidence interval when forecasting RI variations within a 0.01 RIU range. This not only streamlines biosensing protocols by obviating the need for specialized sensor sensitivity calibration but also preserves the efficacy and efficiency of the sensing layer.

In the context of insulin sensor biofunctionalization and biodetection, we conducted comparative analyses with the cross-demodulation technique. The signal achieved through our regressive model enables a precise differentiation between distinct stages, revealing attachment dynamics previously obscured by cross-demodulation. Our filtering methodology captures the entire evolution of the SPR notch, eliminating the need for interpolation. This distinctive feature of our filtering process outperforms the cross-demodulation technique, particularly in cases where spectrum attenuation is not sufficiently pronounced or when spectral alterations are localized at the attenuation edge. In such scenarios, observed for sub-mRIU changes during the insulin detection process (refer to the corresponding insertion losses in Fig. 8), there is an ambiguous shift of the crossed point (red) within the envelope-based demodulation framework. Furthermore, presenting RI evolution, rather than wavelength fluctuations, overcomes the longstanding challenge of sensor comparability.

## Methods

### Sensor fabrication

The fabrication process of Au-TFBGs, which serves as the basis for the biosensors used in this study involves several steps. A silica telecommunication-grade optical fiber (Corning SMF-28) is first hydrogen-loaded at ~200 bar and 60 °C for 30 h in order to enhance its photosensitivity. Then, using the phase mask technique, a tilted Bragg grating is inscribed within the fiber core. An excimer laser (Noria, from Northlab Photonics) operating at 193 nm is used in conjunction with an 8°-tilted phase mask with a period of 1100 nm. To eliminate hydrogen in excess, the fiber is heated at 100 °C for 24 h. Finally, a 50 nm-thick gold layer is deposited on the fiber using a sputter-coater Spuco equipped with 2 in. magnetron modules operating at a 250 W RF power supply. The deposition process is monitored using an inbuilt quartz microbalance with a resolution of 0.1 nm.

### Index solutions

The index solutions were based on LiCl and had varying concentrations covering RI range from 1.3332 RIU (pure water) to 1.3430 RIU for the most concentrated solution. The RI of the solutions was measured at 589 nm using a portable refractometer (Reichert Analytical Instrument, Brix/RI-check). The index solutions were intentionally formulated to exhibit a minimum difference of 2e-4 RIU, ensuring discernible variations suitable for effective model training.

### Constitutive dataset

To obtain the training data, a series of 16 refractometry experiments were conducted on distinct Au-TFBGs. A total of 610 measurements were conducted, with 140 measurements (the entire data from 4 distinct fibers) reserved for the validation phase. The remaining 470 measurements underwent a data augmentation process, multiplying them by a factor of 49. This augmentation resulted in a comprehensive training dataset comprising 23,170 spectra.

### Experimental setup with the OVA

The process of acquiring data entailed employing an OVA (specifically the OVA CTe 4000NF manufactured by Luna Technologies Inc.) in conjunction with an Au-TFBG directly connected in transmission. The TFBG was placed within a trough, enabling submersion of the fiber in aqueous index solutions. The initial polarization state was established following the procedure outlined in “Data acquisition and preprocessing”.

### Experimental setup with the OSA

ILs measurements were acquired using an unpolarized super wideband light source (Amonics ASLD-CWDM-5-B-FA) and an optical spectrum analyzer (OSA, Yokogawa AQ6370). To adjust the input state of polarization, an in-line linear polarizer followed by a polarization controller was positioned upstream of the TFBG. The sensing section of the Au-TFBG was positioned within a trough, allowing the fiber to be immersed in aqueous index solutions. The input polarization state was determined by observing the spectrum during the polarization adjustment process.

### Experimental setup for biosensing

For the functionalization and biodetection experiments, the Au-TFBG sensor is placed within a microfluidic chamber with a cross-section of (3.2 ± 0.7) mm^2^. The chamber, from microfluidic ChipShop GmbH, has a volume of 120 μL and features two inlets and one outlet, allowing for the alternating delivery of different solutions into the chamber. The output flow rate is regulated at 30 μL min^−1^ to maintain a consistent flow rate during the experiments. The solutions are precisely controlled using double diaphragm pumps integrated with a bubble trap and a pulsation damper (Micropumps mp6, mp-bt, and mp-damper from Bartel Mikrotechnik).

## Data Availability

All data that support this study are available from the corresponding author upon reasonable request.

## Code availability

Code to replicate this research can be requested from the corresponding author upon reasonable request.
